# Memory Extinction Entails the Inhibition of the Transcription Factor NF-κB

**DOI:** 10.1371/journal.pone.0003687

**Published:** 2008-11-10

**Authors:** Emiliano Merlo, Arturo Romano

**Affiliations:** Laboratorio de Neurobiología de la Memoria, Departamento de Fisiología, Biología Molecular y Celular, Facultad de Ciencias Exactas y Naturales, Universidad de Buenos Aires, IFIByNE, CONICET, Ciudad Universitaria, Pab. II, Buenos Aires, Argentina; L'université Pierre et Marie Curie, France

## Abstract

In contextual memories, an association between a positive or negative reinforcement and the contextual cues where the reinforcement occurs is formed. The re-exposure to the context without reinforcement can lead to memory extinction or reconsolidation, depending on the number of events or duration of a single event of context re-exposure. Extinction involves the temporary waning of the previously acquired conditioned response. The molecular processes underlying extinction and the mechanisms which determine if memory will reconsolidate or extinguish after retrieval are not well characterized, particularly the role of transcription factors and gene expression. Here we studied the participation of a transcription factor, NF-κB, in memory extinction. In the crab context-signal memory, the activation of NF-κB plays a critical role in consolidation and reconsolidation, memory processes that are well characterized in this model. The administration of a NF-κB inhibitor, sulfasalazine prior to extinction session impeded spontaneous recovery. Moreover, reinstatement experiments showed that the original memory was not affected and that NF-κB inhibition by sulfasalazine impaired spontaneous recovery strengthening the ongoing memory extinction process. Interestingly, in animals with fully consolidated memory, a brief re-exposure to the training context induced neuronal NF-κB activation and reconsolidation, while prolonged re-exposure induced NF-κB inhibition and memory extinction. These data constitutes a novel insight into the molecular mechanisms involved in the switch between memory reconsolidation and extinction. Moreover, we propose the inhibition of NF-κB as the engaged mechanism underlying extinction, supporting a novel approach for the pharmacological enhancement of this memory process. The accurate description of the molecular mechanisms that support memory extinction is potentially useful for developing new strategies and drug candidates for therapeutic treatments of the maladaptive memory disorders such as post-traumatic stress, phobias, and drug addiction.

## Introduction

Since the original interpretation by Pavlov [1], extinction of an associative memory was considered a new process that impedes the expression of the original association. Under this interpretation, the original memory is not abolished by extinction and, in most cases, can be recovered spontaneously or after behavioural or pharmacological treatments [2]–[5]. Such hypothesis suggests the formation of new neuronal circuits underlying the newly acquired behavioural outcome. Several drugs, acting in a limited period after extinction induction, interfere with this process and allow the original long-term memory to be expressed [4], [6]. The fact that protein synthesis inhibitors and NMDA-type glutamate receptors (NMDAR) antagonists are effective drugs affecting extinction led to the hypothesis that this process required consolidation-like mechanisms similar to the consolidation of the original memory [7]–[11]. However, beyond the requirement of *de novo* protein synthesis and NMDAR, some research data point out differences between the molecular mechanisms involved in memory consolidation and extinction, as the participation of protein phosphatases [12], [13] and endocanabinoids [14]. Interestingly, both molecular mechanisms, as well as NMDAR [15], are involved in long-term depression (LTD), a neural plasticity model that induces a reversible reduction of synaptic efficacy, suggesting that synaptic weakening of the original consolidated memory trace can explain in part the neural process involved in memory extinction.

In the core of the molecular mechanisms involved in the long-term persistence of memory trace is the regulation of gene expression, conducted *via* the activation of specific transcription factors (TFs). These mechanisms are considered key molecular processes in consolidation [16]–[18] and reconsolidation [19]–[22]. Related to the role of gene transcription during LTM extinction there is indirect evidences provided by the use of *in vivo* protein synthesis inhibition and the inhibition of protein kinases that are involved in gene regulation. At our knowledge, only two reports evaluate the hypothesis by direct blockade of the transcriptional machinery [23], [24]. One of these reports studied the effect of intrahippocampal injection of the transcription inhibitors alpha-amanitin and DRB on inhibitory avoidance in rats [23]. In that report, the drug infusions impaired memory extinction when administered before the extinction protocol, suggesting that the transcriptional activity is required for consolidation of extinction. Conversely, Lin and colleagues reported that memory extinction is insensitive to actinomycin-D and depends upon calcineurin activity that induces dephosphorylation of cAMP response element binding protein (CREB) [24]. This last finding suggests that CREB inhibition by calcineurin and the consequent gene transcription inhibition could be part of the molecular mechanisms involved in extinction.

In the context-signal memory in crabs [25], the presentation of a danger stimulus (an opaque screen passing over the animal) provokes an escape response that is actively replaced in successive events by a freezing response as a consequence of the repetition of the stimulation [26]. As a consequence of a training session of 15 or more spaced trials, an associative LTM is formed linking the environmental cues (the “context”) with the danger stimulus episode [27]. This context-dependent memory is defined at testing as a significantly lower response to the danger stimulus by a trained group of animals than by an untrained control group. Animals with fully consolidated LTM that are exposed to the training context would either reconsolidate or extinguish LTM depending on the length of the context exposure session [10]. While a brief presentation (5 min) induces reconsolidation, a long period (>1h) induces extinction [10], [28]. In *Chasmagnathus*, LTM consolidation and reconsolidation depends upon *de novo* protein synthesis and the activity of the TF Nuclear Factor kappa B (NF-κB) activity [29]–[31]. Crab's LTM extinction is blocked by protein synthesis inhibitors [10].

The participation of NF-κB in memory consolidation and reconsolidation processes constitutes an evolutionary conserved feature, since it was shown that its activation is a necessary mechanism in crabs and mice [18], [21], [22], [33], [34]. All these evidence locate NF-κB as a key partner among the molecular mechanisms involved in LTM formation and persistence.

In this report we study the role of the TF NF-κB in memory extinction in the crab *Chasmagnathus*. We analyze: 1) the state of activity of NF-κB in the brain during extinction session, and 2) the effect of the inhibition of the TF during extinction training on memory extinction. We found that during extinction induction NF-κB levels of activity are significantly diminished in the brain, and that the pharmacological inhibition of NF-κB during extinction training strengthened memory extinction.

## Results

### Inhibition of NF-κB during extinction session impairs spontaneous recovery

Memory reactivation induced by animal re-exposure to the training context activates NF-κB as part of the molecular mechanisms involved in memory reconsolidation. Such activation was found in different types of contextual memory models as context-signal memory in the crab *Chasmagnathus* [21], contextual fear conditioning in rats [34] and inhibitory avoidance in mice [22]. In the crab model, 5 min of re-exposure to the training context 24 h after learning induces reconsolidation that is impaired with the pre-exposure administration of the NF-κB inhibitor sulfasalazine. Conversely, 2 h re-exposure to the context induces protein synthesis-dependent extinction [10]. This extinction is expressed during testing session as a lack of retention, in spite of the strong training protocol used during the training day.

**Figure 1:**
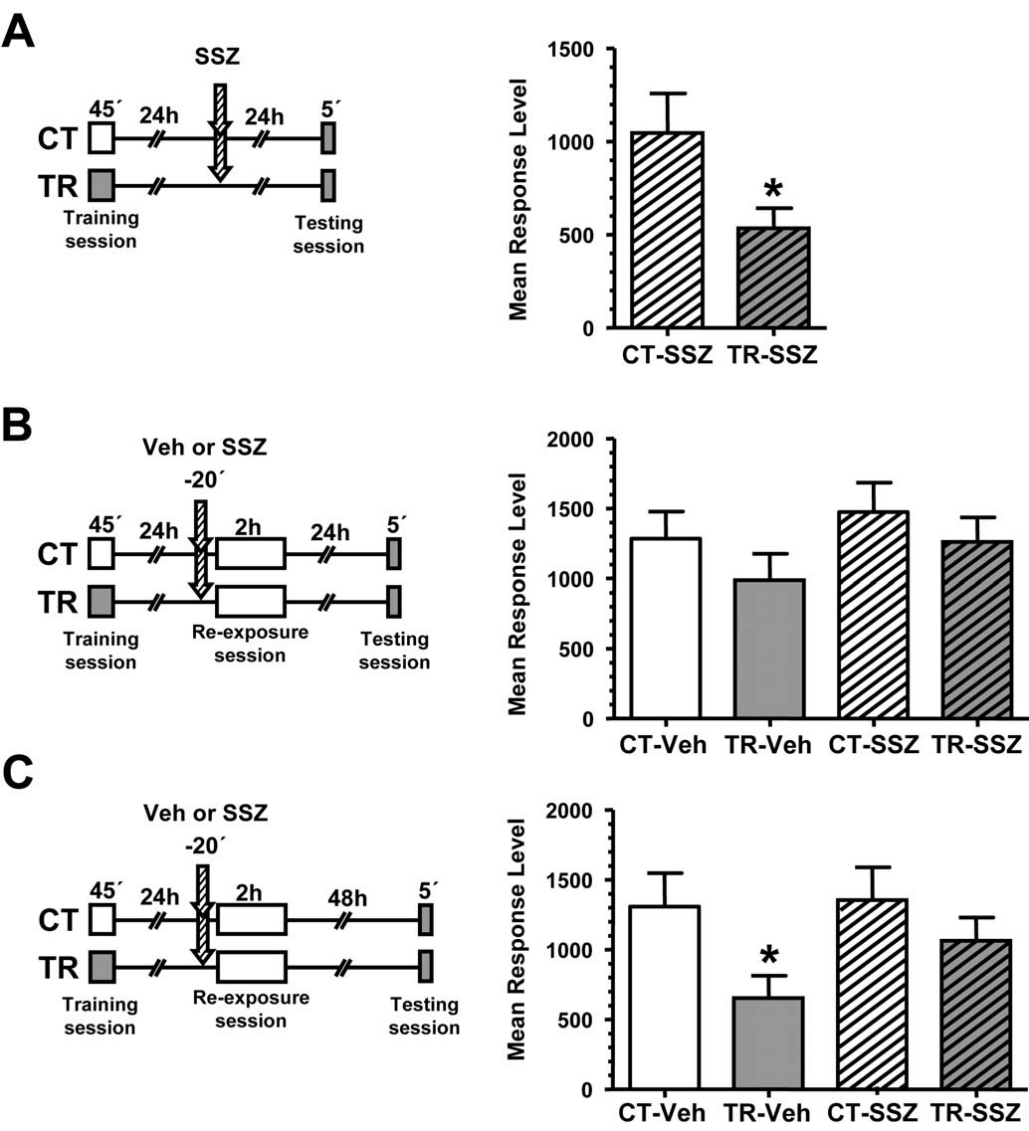
Effect of sulfasalazine on memory extinction. A. Effect of sulfasalazine injection 24 h after training without re-exposure session. *Left panel*: Experimental design. On day 1 the animals were trained with 15 trials (TR groups) or were located in the training apparatus but remained untrained (CT groups). On day two, the animals were injected with 6 μg/g sulfasalazine (indicated with arrows). 24 h after the injection the animals were tested for memory retention with one trial in the training context. *Right graph:* Performance at testing session. B. Effect of sulfasalazine 20 min before prolonged re-exposure session. *Left panel*: On day 1 the animals were trained with 15 trials (TR groups) or were located in the actometers but remained untrained (CT groups). On day two, a pair of CT-TR groups was injected with 6 μg/g sulfasalazine and the other with vehicle solution (indicated with arrows). 20 min later, all groups were re-exposed to the training context for 2 hours. 24 h after the extinction session the groups were tested for memory retention with one trial in the training context. *Right graph:* Performance at testing session. C. Effect of sulfasalazine in spontaneous recovery. *Left panel*: the same as in B, but the testing session was given 48 h after the extinction protocol. *Right graph:* Performance at testing session. Values are Mean Response Level±SEM at testing. *, *p*<0.05.

To assess the eventual effect of NF-κB inhibition on extinction we trained a group of animals (TR group) with 15 trials while a control group (CT group) remained in the experimental device without stimulus presentation (Figure 1a, left diagram). Under such conditions, a long-term association between context features and the danger stimulus is formed [35]. This association is assessed as a significant lower level of response of TR group respect to CT group at testing. Such a low level is an expression of an active change from escape to freezing response [36]. Twenty four h later, the animals were injected in the pericardial sac with 6.6 μg/g sulfasalazine or vehicle 20 min before the extinction session, consisting of a re-exposure to the training context for 2 h. Then, two pairs of CT/TR groups were formed (CT-Veh/TR-Veh and CT-SSZ/TR-SSZ). A third pair of CT/TR groups injected with sulfasalazine but not re-exposed to the training context was used in order to evaluate the effect of the drug 24 h after training without the extinction session (Figure 1a). Sulfasalazine is a specific inhibitor of IκB kinase (IKK), the kinase that phosphorylates and induces the degradation of IκB, the inhibitor of NF-κB. The used dose is effective to induce an inhibition of about 50% in basal NF-κB activity for 45 min and to impair memory consolidation and reconsolidation [18], [21]. One day after the injection and the extinction protocol, the testing session showed, as expected, memory retention for the trained group without exposure (F _(1,74)_  =  5,52; *p*<0.05, CT-SSZ vs. TR-SSZ, Figure 1a). This result confirms previous results showing that sulfasalazine has no effect administered 24 h after training without re-exposure to the training context. Conversely, no memory retention was found for the two trained groups that were injected with vehicle or sulfasalazine and then re-exposed to the training context (no significant differences between CT and TR pair of groups, Figure 1b). These results indicate memory extinction for both groups re-exposed to the training context for 2 h. At variance with the effect of the protein synthesis inhibitor cychoheximide [10], sulfasalazine injection did not impede extinction, suggesting at first glance that NF-κB activity is not required for consolidation of extinction.

In the crab model, spontaneous recovery of memory retention occurs between 2 and 3 days after extinction induction (Hepp Y, Pedreira ME, personal communication). In order to study the effect of sulfasalazine on spontaneous recovery of extinction, we performed the following experiment with the same design of the previous one but with 48 h interval between extinction session and testing, instead of 24 h (Figure 1c, left diagram). In this instance, we found retention in the vehicle-injected trained group (F _(1,156)_  =  5,19; *p*<0.05, CT-Veh vs. TR-Veh) due to spontaneous recovery, but no retention in the sulfasalazine trained group (F _(1,156)_  =  1,03; *p*>0.05, between CT-SSZ and TR-SSZ groups) (Figure 1c, graph on the right). These results suggest that NF-κB inhibition facilitates extinction, impairing the spontaneous recovery of the original memory trace.

### NF-κB inhibition facilitates extinction

Although the results of the previous experiment are in agreement with extinction facilitation, the lack of retention in sulfasalazine-injected group can also be explained in terms of impairment of the original memory trace induced during memory reactivation by retrieval. As shown in the previous section, sulfasalazine does not impair memory when injected 24 h after training without context re-exposure but shows amnesic effect if it is administered prior to 5 min context re-exposure. Sulfasalazine inhibits NF-κB activation induced by retrieval [21]. Thus, in the last experiment (Fig 1c) memory may become labile after retrieval induced by context re-exposure. However, in previous studies on this model, Pedreira and Maldonado demonstrated, using cycloheximide, that the original memory remained unaffected after a long re-exposure of 1 or 2 h, i.e., when extinction was induced. In that circumstance, extinction was impeded by protein synthesis inhibition [10]. Those findings supported the idea that reconsolidation and extinction are not coexisting, so that the original memory cannot be disrupted by drugs when an extinction process is ongoing. We performed the following experiments in order to evaluate these two alternative interpretations for sulfasalazine effect: extinction facilitation or original memory impairment.

**Figure 2:**
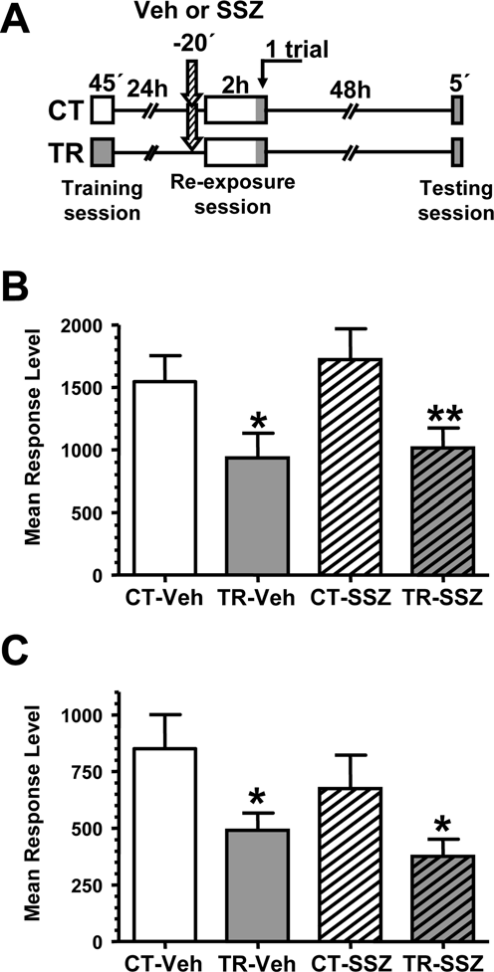
The effect of sulfasalazine injection in memory extinction is cancelled by US presentation during CS re-exposure. A. Experimental design: On day 1 the animals were trained with 15 trials (TR groups) or were located in the training apparatus but remained untrained (CT groups). On day two, a pair of CT-TR groups was injected with 6 μg/g sulfasalazine and the other with vehicle solution (indicated with arrows). 20 min later, all groups were re-exposed to the training context for 2 hours. At the end of the 2h context re-exposure one trial was presented. 48 h after the extinction session the groups were tested for memory retention with one trial in the training context. B. Performance elicited by the trial at the end of the extinction session. C. Performance at testing session. Values are Mean Response Level±SEM at testing. *, *p*<0.05.

In previous studies on this model, context re-exposure of 2 h failed to induce extinction when the danger stimulus was presented at the end of the extinction session, before animals are removed from the training context [28]. This finding prompted us to the following proposition: If sulfasalazine injection disrupts the original memory, no retention should be found even after a stimulus presentation at the end of the 2 h re-exposure session. Taking advantage of this tool, it could be possible to dissect lack of retention due to extinction facilitation from that due to original memory impairment. Thus, in the following experiment the same design was used but we included one stimulus presentation at the end of the extinction session (Fig 2a). As expected on the basis of previous studies [28], we found that the stimulus presentation impeded short-term and long-term extinction in the TR-Veh group (Figure 2b and c) (CT-Veh vs. TR-Veh, F _(1,135)_  =  3,93, *p*<0.05 and F _(1,135)_  =  5,70, *p*<0.05, respectively). Similar results were found in the sulfasalazine injected groups (Fig 2b and c) (CT-SSZ vs. TR-SSZ, F _(1,135)_  =  5,03, *p*<0.05 and F _(1,135)_  =  4,72, *p*<0.05, respectively). Thus, these findings suggest that the original memory was not affected by sulfasalazine administration prior to extinction session.

**Figure 3:**
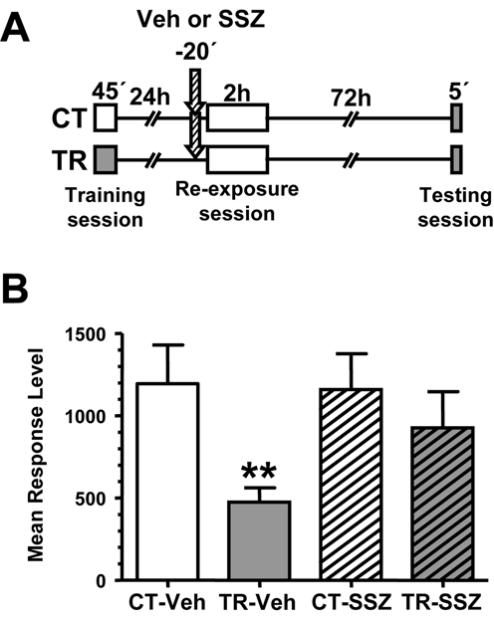
The effect of sulfasalazine injection in memory extinction endures for at least 72 h. A. Experimental design: On day 1 the animals were trained with 15 trials (TR groups) or were located in the training apparatus but remained untrained (CT groups). On day two, a pair of CT-TR groups was injected with 6 μg/g sulfasalazine and the other with vehicle solution (indicated with arrows). 20 min later, all groups were re-exposed to the training context for 2 hours. 72 h after the extinction session the groups were tested for memory retention with one trial in the training context. B. Performance at testing session. Values are Mean Response Level±SEM at testing. *, *p*<0.05.

In order to obtain further evidence, we performed an experiment in which an extinction session is presented 24 h after training and the testing session was delayed from 48 h to 72 h (Figure 3a, upper diagram). A delay in the spontaneous recovery of the original memory may be expected if sulfasalazine induces extinction facilitation. However, for longer periods between extinction session and testing the original memory should be recovered. As expected, the analysis of the Veh pair of groups showed memory recovery (CT-Veh vs. TR-Veh, F _(1,154)_  =  6,62, *p*<0.05) but in the analysis of sulfasalazine injected groups no retention was found (Figure 3b). Thus, sulfasalazine impedes memory recovery even 72 h after the extinction session.

**Figure 4:**
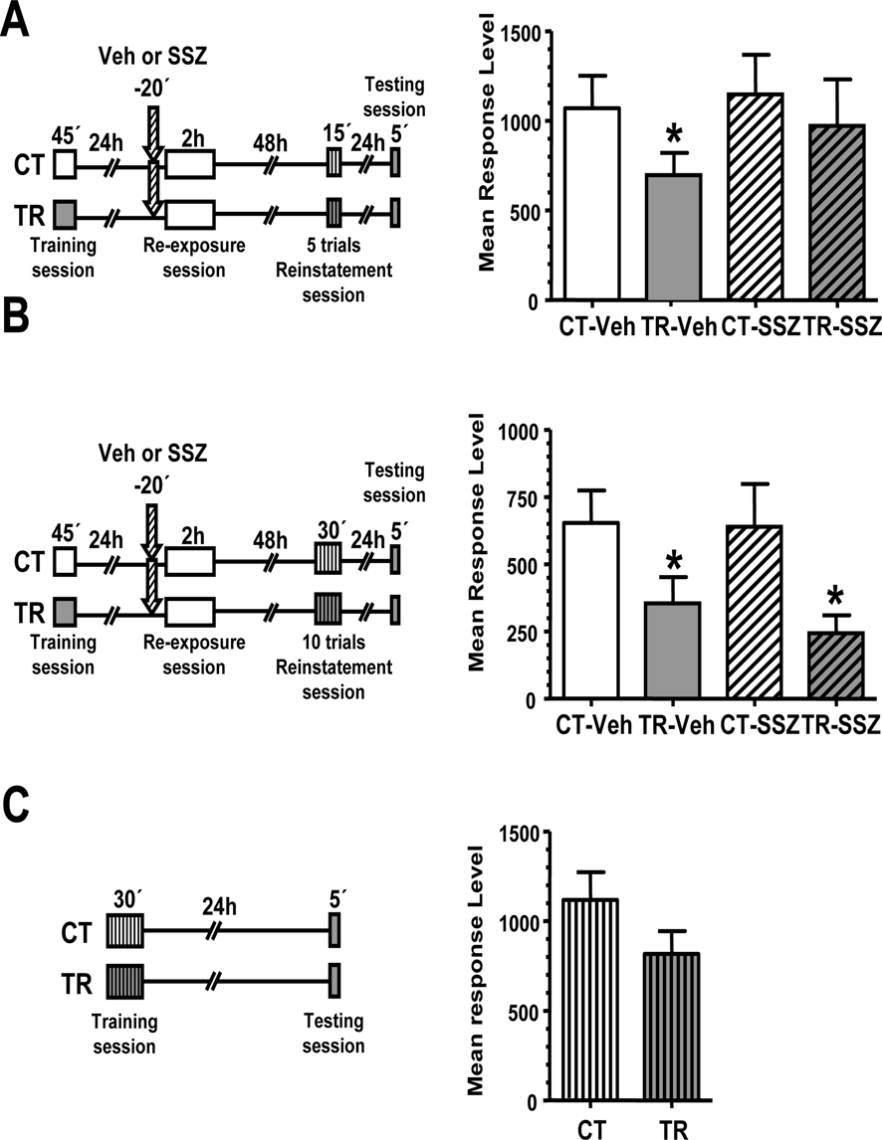
Reinstatement reveals the original memory over sulfasalazine-facilitated memory extinction. A. The reinstatement with 5 trials fails to reveal the original memory over sulfasalazine-facilitated memory extinction. *Left panel:* Experimental design. On day 1 the animals were trained with 15 trials (TR groups) or were located in the training apparatus but remained untrained (CT groups). On day two, a pair of CT-TR groups was injected with 6 μg/g sulfasalazine and the other with vehicle solution (indicated with arrows). 20 min later, all groups were re-exposed to the training context (context A) for 2 hours. 48 h after the extinction session the groups were reinstated with 5 trials in a different context (B). 24 h later all groups were tested for memory retention with one trial in the training context. *Right graph:* Performance at testing session. B. The reinstatement with 10 trials reveals the original memory over sulfasalazine-facilitated memory extinction. *Left panel:* Experimental designs in A but using 10 trials during the reinstatement session. *Right graph:* Performance at testing session. C. 10 trials training is insufficient to induce long-term memory in a novel context. *Left panel:* Experimental design. On day 1 the animals were trained with 10 trials (TR groups) or were located in the context B but remained untrained (CT groups). 24 h later, all groups were exposed to the context A and tested for memory retention with one trial. *Right graph:* Performance at testing session. Values are Mean Response Level±SEM at testing. *, *p*<0.05. Plain box: context A. Stripped box: context B.

In the third experiment of this section we explored if it is possible to induce the recovery of the original memory by means of reinstatement [2], [37] in SSZ treated animals. For this purpose, we introduced a session of 5 danger stimulus presentations in a different context (context B) 48 h after the extinction session and 1 day before testing performed in the normal context (context A) (Figure 4a left panel). Figure 4a shows memory recovery in the Veh pair of groups (CT-Veh vs. TR-Veh, F _(1,131)_  =  4,45, *p*<0.05) but not in the SSZ pair of groups, indicating that 5 trials of danger stimulus are not able to reinstate the original memory in the drug treated group.

Next, we repeated the previous experiment but including 10 trials (instead of 5) of reinstatement (Figure 4b, left panel). As shown in the graph of Figure 4b, the stronger reinstatement session was able to recover memory retention in both Veh and SSZ pairs of groups (CT-Veh vs. TR-Veh, F _(1,132)_  =  4,58, *p*<0.05; CT-SSZ vs. TR-SSZ, F _(1,132)_  =  6,30, *p*<0.05).

Finally, in order to rule out the possibility that 10 trials in a different context are able to induce long-term memory retention *per se*, we included an experiment in which a group of animals was trained in context B with 10 trials and the other group was exposed to context B but remained untrained. The following day, animals were tested in context A and no statistically significant differences were found between groups (Fig 4c, left panel), indicating that 10 trials were not enough to induce significant retention. This result is expected taken into account that 15 trials, but not 10 trials, are required to induce long-term memory in this model [27], and that this memory is context-specific [35], [38]. Thus, the retention found in the previous experiment was due to memory reinstatement from extinction, indicating the presence of the original memory.

The experiments of the present section support that the retention deficit induced by sulfasalazine is due to extinction facilitation and not to impairment of the original memory.

### NF-κB is inhibited during extinction session

In the following experiments we measured nuclear NF-κB activity during extinction induction. For this purpose nuclear extracts were obtained from the central brain at different time points during extinction session. With these extracts we performed electrophoretic mobility shift assay (EMSA), a technique that allow to determine changes in the specific NF-κB DNA binding activity and to detect the presence or absence of this TF in nuclei [32].

**Figure 5:**
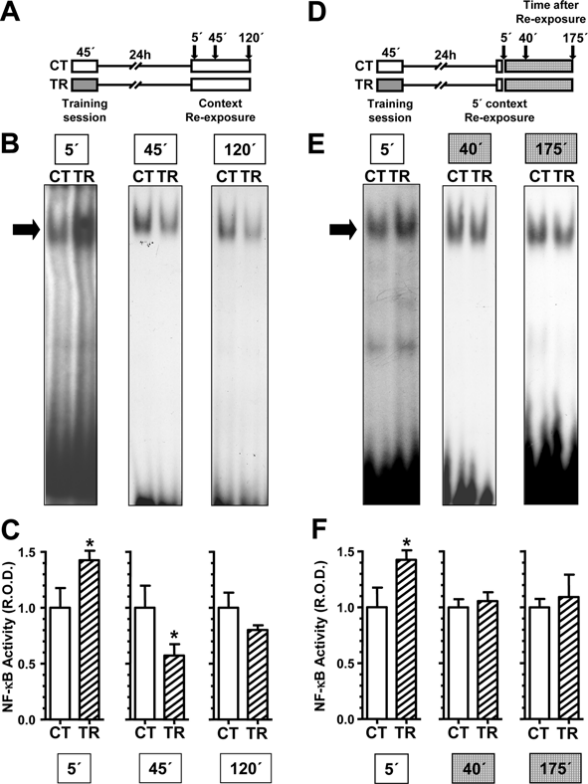
NF-κB activity during extinction session. A. Top diagram: experimental design. On day 1 the animals were trained with 15 trials (TR groups) or were located in the training apparatus but remained untrained (CT groups). On day two, the CT-TR groups were re-exposed to the training context. At different time points (indicated by arrows), animals of CT and TR pair of groups were anesthetized and nuclear extracts from the 20 central brains of each group were obtained. B. Representative EMSAs performed with nuclear extracts obtained from CT and TR groups at 5, 45 and 120 minutes of context re-exposure. The specific band is indicated with a filled arrow. C. NF-κB activity in TR group relative to CT. Mean±SEM of relative optic density (ROD) values of the specific NF-κB retarded band normalized to CT group media, obtained in four independent experiments. D, E and F. NF-κB activity after memory reactivation. D. Top diagram: experimental design. On day 1 the animals were trained with 15 trials (TR groups) or were located in the training apparatus but remained untrained (CT groups). On day two, the CT-TR groups were re-exposed for 5 minutes to the training context. At different time points after the re-exposure (indicated by arrows), animals of CT and TR pair of groups were anesthetized and nuclear extracts from the 20 central brains of each group were obtained. E. Representative EMSAs performed with nuclear extracts obtained from CT and TR groups at 5, 45 and 180 minutes after context re-exposure. The specific band is indicated with a filled arrow. F. NF-κB activity in TR group relative to CT. Mean±SEM of relative optic density (ROD) values of the specific NF-κB retarded band normalized to CT group media, obtained in four independent experiments. *, *p*<0.05 in *t-*test.

In the first experiment, TR group received a 15 trials training and CT group remained in the training chamber without stimulation. Twenty four h later, each group was divided in three groups of 20 crabs that were re-exposed to the training context and remained there either for 5, 45 or 120 min. Animals were then anesthetized in ice-cold water, sacrifice, and the central brain was dissected (Fig 5a). We obtained nuclear extracts from the pool of 20 central brains for each group and we performed EMSAs with a κB consensus sequence DNA oligonucleotide as a probe. Using this probe one retarded band is observed. This band is specific for NF-κB, as previously demonstrated [39], [32]. Four independent experiments were performed and the densitometric values of this band were analyzed as relative NF-κB activity to the respective CT group (Fig 5b and c). As we had previously found [21], a 5 min re-exposure induced memory reconsolidation and NF-κB activation (*p*<0.05, *t*-test). Conversely, after a 45 min re-exposure to the training context NF-κB was inhibited (*p*<0.05, *t*-test) and this inhibition persisted, although with less intensity and without significant differences after 120 min (Fig 5b and c). These time points are coincident with the induction of memory extinction [10]. However, if animals were re-exposed to the training context for 5 min and then were removed and placed in individual containers (Fig 5d), the initial activation of NF-κB decreased to basal level at 40 or 175 min (Fig 5e and f). Thus, at variance with NF-κB inhibition found when animals remained for a prolonged period in the training context, no inhibition was found when the crab were exposed for only 5 min and then removed. These data support that NF-κB is specifically inhibited during the induction of extinction but not by the mere time course.

### Memory deficit induced by NF-κB inhibition in reconsolidation can be recovered by reinstatement treatment

**Figure 6:**
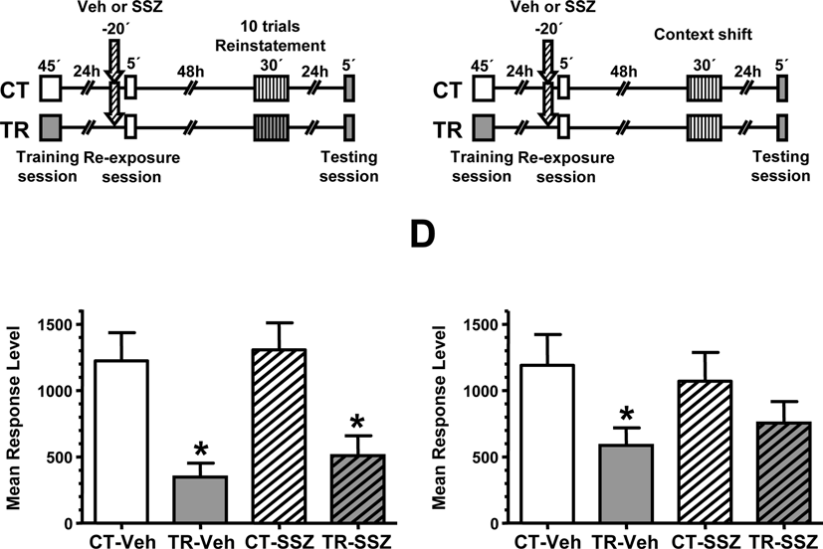
The reinstatement with 10 trials reveals the original memory over sulfasalazine-induced amnesia in memory reconsolidation. A. Experimental design: On day 1 the animals were trained with 15 trials (TR groups) in the context A or were located in the context but remained untrained (CT groups). On day two, a pair of CT-TR groups was injected with 6 μg/g sulfasalazine and the other with vehicle solution (indicated with arrows). 20 min later, all groups were re-exposed to the context A for 5 minutes. 48 h after re-exposure session the groups were reinstated with 10 trials in the context B. 24 h later all groups were tested for memory retention with one trial in the context A. B. Performance at testing session. C. Experimental design: On day 1 the animals were trained with 15 trials (TR groups) in the context A or were located in the context but remained untrained (CT groups). On day two, a pair of CT-TR groups was injected with 6 μg/g sulfasalazine and the other with vehicle solution (indicated with arrows). 20 min later, all groups were re-exposed to the context A for 5 minutes. 48 h after re-exposure session the groups were exposed to the context B for 30 minutes. 24 h later all groups were tested for memory retention with one trial in the context A. D. Performance at testing session. Values are Mean Response Level±SEM at testing. *, *p*<0.05.

Memory activation induced by animal re-exposure to the training context for a short time (i.e., 5 min) induces NF-κB activation and reconsolidation. When such activation is blocked by sulfasalazine, a retention deficit is found [21]. On one hand, sulfasalazine administration at the same dose used in the present work induces NF-κB inhibition of about 50% on basal activity during 45 min [18]. On the other hand, the last results of the present work showed that NF-κB inhibition is part of the molecular processes of extinction induction. Bearing these two facts in mind, we asked whether sulfasalazine during the reconsolidation protocol could induce memory extinction by prolonged NF-κB inhibition during memory reactivation rather than by disrupting memory reconsolidation. If this is the case, a reinstatement protocol should induce memory recovery. On the contrary, if sulfasalazine provokes long-term memory impairment the reinstatement treatment should not be able to induced memory recovery. To test these two alternatives, we performed a reconsolidation experiment in which, one day after the training session, animals were injected with vehicle or sulfasalazine and after 20 min were re-exposed to the training context for 5 min. Forty eight h later, a reinstatement protocol of 10 trial of stimulus presentation in context B was performed. A testing session was then presented 24 h after the reinstatement session. Two pairs of groups without reinstatement session were included to assess the effect of sulfasalazine in reconsolidation, as previously found [21] (Fig 6a and c). Both pairs of reinstated groups, CT-Veh/TR-Veh and CT-SSZ/TR-SSZ showed retention (CT-Veh vs. TR-Veh, F _(1,156)_  =  12,85, *p*<0.01; CT-SSZ vs. TR-SSZ, F _(1,156)_  =  11,02, *p*<0.05, Fig 6b) while among the groups without reinstatement session only CT-Veh/TR-Veh pair showed retention (CT-Veh vs. TR-Veh, F_(1,152)_ =  5,06, *p*<0.05), and no retention was found in the sulfasalazine CT vs. TR groups comparison (Fig 6d). These results indicate that the retention deficit induced by sulfasalazine in reconsolidation can be recovered by means of reinstatement treatment.

## Discussion

**Figure 7:**
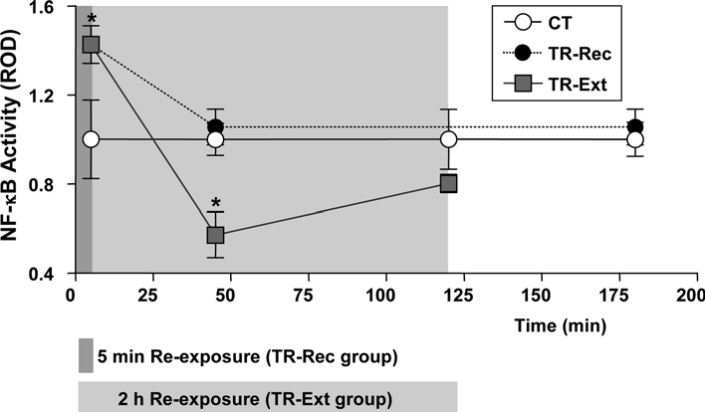
Time course of NF-κB activity underlying memory reconsolidation or extinction. Summary of EMSA experiments shown in Fig. 5. CT, control group; TR-Rec, trained group re-exposed for 5 minutes to the training context; TR-Ext, trained group re-exposed 5, 45 or 120 minutes to the training context. Mean±SEM of relative optic density (ROD) values of the specific NF-κB retarded band normalized to CT group media, obtained in four independent experiments for each time point and treatment. *, *p*<0.05 in *t-*test.

**Figure 8:**
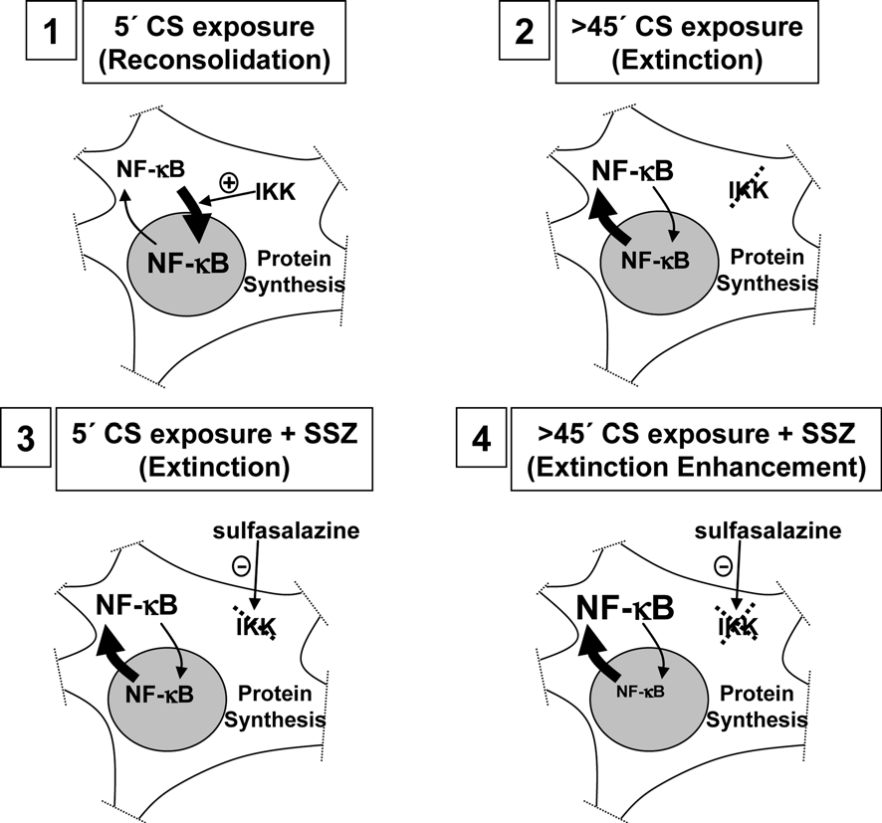
A model of NF-κB role in memory extinction and reconsolidation, and the effect of its inhibition. 1) Five min context re-exposure induces NF-κB-dependent and protein synthesis-dependent memory reconsolidation. 2) Prolonged re-exposure induces NF-κB inhibition and protein synthesis-dependent memory extinction. 3) NF-κB inhibition by sulfasalazine plus 5 min re-exposure mimics memory extinction. 4) NF-κB inhibition by sulfasalazine plus prolonged re-exposure induces extinction facilitation.

The findings of the present work provide evidence about the role of the TF NF-κB in memory extinction and new insights on its role in reconsolidation. We initially found that the NF-κB inhibitor sulfasalazine produce a lack of spontaneous recovery that normally occurs 2 or 3 days after the induction of extinction. A series of experiments using sulfasalazine in the extinction session supports that NF-κB inhibition induced extinction facilitation but not the impairment of the original memory. On the one hand, the retention deficit induced by sulfasalazine was not found when extinction was impeded by the danger stimulus presentation at the end of the extinction session (Fig 2a). On the other hand, a reinstatement protocol rescued the retention deficit provoked by sulfasalazine administered previous to the extinction protocol. In agreement with these findings, the time course of NF-κB activity in the central brain after memory retrieval showed that, initially, activation occurred but was followed by inhibition with prolonged re-exposure to the training context, a treatment that induces memory extinction. If animals were re-exposed only for 5 min, a protocol that induces reconsolidation, and then placed in another context for the rest of the time before NF-κB activity determination, no inhibition was found, but only the initial activation (Fig 7). These findings are interpreted in the following terms: retrieval induced by re-exposure to the training context provokes NF-κB activation as part of the molecular processes required for long-term memory re-stabilization. However, in coincidence with the switch between reconsolidation and extinction [10], the prolonged exposure to the CS (training context) without reinforcement initiate a process that leads to NF-κB inhibition as part of the molecular mechanisms of extinction (Fig 8).

The long-term persistence of memory requires regulation of gene expression and, particularly, activation of the NF-κB pathway. Inversely, extinction that involves the temporary waning of the conditioned response would imply, among other mechanisms, exportation of NF-κB from the nucleus with the subsequent prevention of NF-κB transcriptional activity.

On the basis of the present findings and previous results, we propose a working model for NF-κB role in memory after retrieval (Fig 8). The initial process of transcriptional activation induced by retrieval would be mediated by protein kinases. In particular, the activation of IKK protein kinase induces NF-κB translocation to the nucleus and its activation. The prolonged presence of the CS would induce activation of other mediators such as protein phosphatases (i.e. calcineurin) that can increase the level of NF-κB inhibitor IκB and produce its nuclear exportation [40]. Under this interpretation, the administration of sulfasalazine during memory reactivation reinforced the effect of prolonged exposure to the CS, provoking extinction strengthening. Accordingly, in the last experiment of the present paper (Fig 6b), the retention deficit induced by sulfasalazine administered before memory reactivation, is reverted by a reinstatement session because the temporary inhibition of NF-κB mimicked the extinction process, allowing a short 5 min exposure to the context to induce memory extinction.

Our interpretation is in line with the point of view that weakening of the original consolidated circuit is part of the neural correlate of memory extinction. However, we cannot exclude the requirement of reinforcement mechanisms in other circuits, non-dependent of NF-κB pathway, mediating the same extinction process. For instance, in other memory model, fear conditioning in rats, the medial prefrontal cortex is involved in extinction by feed-forward inhibition in the lateral amygdala [41]. In such a case, c-fos immediate early genes expression was reported [42], indicating activation of TFs and gene expression.

Recent reports shed light into the molecular mechanisms engaged during memory extinction. Lin and co-workers found that in mice amygdala, Ca^2+^-dependent phosphatase calcineurin levels are rapidly increase during extinction induction (a process that required protein synthesis), and that extinction is blocked after pharmacological inhibition of calcineurin [24]. Moreover, a correlation was established between the extinction protocol and a decrease in phosphorylated CREB levels, the active form of CREB. In the same direction, it was shown that the extinction of fear memory can be facilitated by the transgenic inhibition of PKA, a well known pathway to CREB activation [43]. On the basis of these previous reports and the present work, we propose that extinction of long-term memories depends upon the inhibition of the transcriptional machinery engaged during the original memory trace consolidation. In other words, the inhibition of gene expression could be a necessary step in order to extinguish long-term memories. In several memory models in both vertebrates and invertebrates extinction requires *de novo* protein synthesis [4], [8], [10]. The evidence presented here support that extinction does not required the activity of a key TF involved in consolidation and reconsolidation but actually, requires its inhibition. Similarly, LTD, a model of neural plasticity that involves synaptic weakening, is also dependent on protein synthesis but not on gene transcription [44].

In most cases, extinction is less persistent than the original memory, a fact that allows for spontaneous recovery of the original memory trace. Since gene expression is necessary for persistence of long-lasting neural plasticity, the inhibition of TFs and the concomitant transcription inhibition during extinction could explain in part the less persistent nature of extinction memory.

It is noteworthy that NF-κB is involved in memory consolidation and reconsolidation both in crabs and rodents [22], [31], [34], suggesting that its role is evolutionarily conserved. Here we described in the crab *Chasmagnathus* memory system that inhibition of this TF enhanced memory extinction. Taking into account that pharmacological interventions to enhance extinction have been proposed as potential approaches to the treatment of maladaptive memory disorders [45]–[48], the elucidation of novel molecular mechanisms underlying extinction could help to identify novel molecular candidates for pharmacological interventions that may provide more effective treatments of such neuropsychiatric disorders.

## Materials and Methods

### Animals

Adult male *Chasmagnathus granulatus* intertidal crabs, 2.6–2.9 cm across the carapace, weighing 17±0.2 g (n = 60), were collected from water less than 1 m deep in the estuarine coasts of San Clemente del Tuyú, Argentina, and transported to the laboratory, where they were lodged in plastic tanks (30×45×20 cm) filled to 0.5 cm depth with diluted (12‰, pH 7.4–7.6) marine water (prepared from Cristalsea Marinemix salts, USA), to a density of 20 crabs per tank. The holding room was maintained on a 12 h light-dark cycle (light on 07:00–19:00 h). Animals were fed rabbit pellets (Nutrientes S.A., Argentina) every 3 days and water was changed after feeding. Temperature of both holding and experimental rooms was maintained within a range of 22–24°C. Experiments were carried out between the third and the tenth day after the animal's arrival. Each crab was used in only one experiment. Experiments were carried out in accordance with the National Institute of Health Guide for the Care and Use of Laboratory Animals (NIH publication 80-23/96), USA, and local regulations. All efforts were made to minimize animal suffering and to reduce the number of animals used.

### Training-testing apparatus

The experimental unit was described in detail elsewhere [49]. Briefly, it consists in a bowl-shaped plastic container where the crab is lodged and an opaque rectangular screen moves horizontally over the animal. Screen displacements evoke a crab's running response and, as a consequence, container vibrations which induce electrical signals through a piezoelectric transducer. Signals recorded during a trial were translated into numerical units ranging from 0 to 8000. The experimental room had 40 units, separated from each other by partitions. A computer was employed to program trial sequences, trial duration and inter-trial intervals, as well as to monitor experimental events.

### Drugs and injection procedure

5-[4-(2-Pyridylsulfamoyl)phenylazo]salicylic acid (sulfasalazine) (Sigma, USA) was freshly dissolved in crustacean saline [50] with 10 mM HEPES pH 7.6 plus 15% dimethylsulfoxide (DMSO), final pH 7.6. Fifty μl of vehicle or drug solution were given through the right side of the dorsal cephalothoracic-abdominal membrane by means of a syringe fitted with a sleeve to control depth of penetration to 4 mm, thus ensuring that the injected solution was released roughly at the centre of the pericardial sac. The total volume of hemolymph was estimated at 5 ml (30 % of the body weight) [51] resulting an approximate 100-fold dilution of the drug in hemolymph.

### Procedure in memory evaluation experiments

Each trial lasted 9 sec and consisted of two cycles of presentation of the screen over the actometer. Each cycle lasted 2.5 sec with 2 sec of interval between cycles. The crab's activity was recorded during the entire trial time.

Each experiment lasted between 3 to 5 days and included three phases, namely, training session (day 1), exposure session (day 2), and testing session (day 3, 4 or 5). Crabs were individually housed during the inter-session interval in plastic containers, covered to a depth of 0.5 cm with marine water and kept inside dimly lighted drawers. The training session consisted of 15 trials with an inter-trial interval of 171 sec. In the exposure session, depending on the protocol, the animals were exposed for 5 min or 2 h to the training context (i.e., container with plain walls) without visual danger stimulus presentation. The testing session consisted of one trial. Both the training and testing sessions were preceded by 15 min of adaptation in the apparatus. The unit used during training session is referred to as the training context. In all experiments, one group was trained (TR-group) while the other was located in the device but remained untrained (control group, CT-group).

When indicated, a pair of CT-TR group was injected with vehicle (Veh-pair) or sulfasalazine (SSZ-pair) 20 minutes before the exposure session. Each experiment consisted of four groups (n  =  30–40 for each group).

In the reinstatement experiments we used two different contexts to train and reinstate the animals. The context A, the training context, consisted of a container with plain orange walls. The context B, the reinstatement context, consisted of a container with black and white striped walls. With such a change in this highly-contrasted visual trait was demonstrated that long-term memory is context-specific [35].

### Data analysis and drug effect evaluation

Retention of learning acquired during training was considered when a statistically lower level of response in the testing session was found for trained group relative to control group injected with the same solution (drug or vehicle). The rationale for this criterion is that in previous experiments performed in our laboratory, a significant difference (*t* test, α = 0.05) between trained (TR) and untrained groups (CT) was invariably disclosed at testing session 24 h or more after training when 15 or more training trials (ITI = 171 sec) were given. Such significant differences were also found when crabs were injected with vehicle at diverse pre- and post-training intervals. Accordingly, predictions are for a significant difference at testing between CT and TR groups. Therefore, throughout this papers results of the behavioural study are analyzed with *a priori* planned comparisons using a weighted means ANOVA with α (per comparison error rate)  =  0.05, according to the standard method [52]. The lack of difference between CT and TR groups is thus assumed as no memory retention. In the case that the extinction protocol is presented, the lack of retention is considered as the extinction memory. A comparison between control groups injected with drug or vehicle was necessary in order to determine eventual drug side-effects affecting the response level at testing in a way not related to training experience. In general, the statistical analysis of testing data included a set of three *a priori* planned comparisons, namely CT-Veh vs. TR-Veh, CT-SSZ vs. TR-SSZ and CT-Veh vs. CT-SSZ, using a weighted means ANOVA with α (per comparison error rate) <0.05 [52], [53]. In the first comparison, a difference between CT-Veh vs. TR-Veh groups was expected due to the reduction in response level induced by training in the latter group. On the contrary, in the second comparison, if the drug impairs retention or spontaneous recovery, no difference was expected between CT-SSZ and TR-SSZ. Finally, as long as the drug does not affect the level of response at testing, no difference was expected in the comparison between control groups.

### Electrophoretic mobility shift assay

Immediately after re-exposure animals were anaesthetized by immersion on ice-cold water for two min. The central brain (supraesophageal ganglion) was then dissected. Twenty ganglions per sample were pooled in 1 ml buffered crab saline solution (pH 7.6). Nuclear extracts were obtained as described previously [32]. To assess NF-κB activity, 12 μg of nuclear proteins extracts were used and double-stranded oligonucleotide DNA containing the NF-κB binding site (5′-AGTTGAG**GGGACTTTCC**CAGGC-3′, binding site in bold) (Promega) was used as probe. With this probe, a single and specific retarded band is found [33]. The relative optical density (R. O. D.) of the band was estimated using NIH ImageJ 1.36b software. All measures were made with exposures within the linear range of the film (Agfa CP-BU). Images were digitalized by means of a scanner for negatives (Umax PowerLook III). Protein contents of the extracts were measured in triplicate by Bradford method and checked for quality and quantity by comparing pattern intensities in SDS-PAGE. CT vs. TR group comparisons was performed by *t*-test.
